# Cerebrospinal Fluid Biomarkers for Diagnosis and the Prognostication of Acute Ischemic Stroke: A Systematic Review

**DOI:** 10.3390/ijms241310902

**Published:** 2023-06-30

**Authors:** Anant Naik, Olufunmilola Adeleye, Stefan W. Koester, Ethan A. Winkler, Joelle N. Hartke, Katherine Karahalios, Sandra Mihaljevic, Anupama Rani, Sudhanshu Raikwar, Jarrod D. Rulney, Shashvat M. Desai, Lea Scherschinski, Andrew F. Ducruet, Felipe C. Albuquerque, Michael T. Lawton, Joshua S. Catapano, Ashutosh P. Jadhav, Ruchira M. Jha

**Affiliations:** 1 Carle Illinois College of Medicine, University of Illinois Urbana-Champaign, Champaign, IL 61820, USA; 2 Mayo Clinic Alix School of Medicine, Mayo Clinic Arizona, Scottsdale, AZ 85259, USA; 3 Department of Neurosurgery, Barrow Neurological Institute, St. Joseph’s Hospital and Medical Center, Phoenix, AZ 85013, USA

**Keywords:** biomarkers, cerebrospinal fluid, genomics, inflammatory markers, ischemic stroke

## Abstract

Despite the high incidence and burden of stroke, biological biomarkers are not used routinely in clinical practice to diagnose, determine progression, or prognosticate outcomes of acute ischemic stroke (AIS). Because of its direct interface with neural tissue, cerebrospinal fluid (CSF) is a potentially valuable source for biomarker development. This systematic review was conducted using three databases. All trials investigating clinical and preclinical models for CSF biomarkers for AIS diagnosis, prognostication, and severity grading were included, yielding 22 human trials and five animal studies for analysis. In total, 21 biomarkers and other multiomic proteomic markers were identified. S100B, inflammatory markers (including tumor necrosis factor-alpha and interleukin 6), and free fatty acids were the most frequently studied biomarkers. The review showed that CSF is an effective medium for biomarker acquisition for AIS. Although CSF is not routinely clinically obtained, a potential benefit of CSF studies is identifying valuable biomarkers from the pathophysiologic microenvironment that ultimately inform optimization of targeted low-abundance assays from peripheral biofluid samples (e.g., plasma). Several important catabolic and anabolic markers can serve as effective measures of diagnosis, etiology identification, prognostication, and severity grading. Trials with large cohorts studying the efficacy of biomarkers in altering clinical management are still needed.

## 1. Introduction

Stroke is a clinical syndrome defined as a sudden loss of neurological function due to a disruption in cerebral blood flow and is a leading cause of disability in the United States [1]. In 2019, the Centers for Disease Control and Prevention identified stroke as the country’s fifth leading cause of death [2]. Stroke can be ischemic or hemorrhagic. Acute ischemic stroke (AIS) accounts for the vast majority (60–80%) of stroke incidence [3]. Strong evidence indicates that early diagnosis and management in patients with AIS are paramount in improving survival and recovering functionality [4,5]. Quick and relatively inexpensive molecular biomarkers have proven to be pivotal in the management of certain diseases, such as troponin use in myocardial infarction or HbA1c in hyperglycemia and diabetes. To this end, identifying AIS biomarkers to accurately diagnose ongoing or impending brain ischemia or infarction and to predict outcomes would be revolutionary in guiding clinical decision-making, improving survival, and limiting disability.

At present, no diagnostic or prognostic molecular marker in AIS has been identified that demonstrates adequate sensitivity, specificity, precision, and cost-effectiveness. In AIS, blood-based biomarker candidates have been extensively researched [6,7]; however, the role of cerebrospinal fluid (CSF) biomarkers has not yet been well elucidated. One challenge associated with using CSF biomarkers is that CSF is not routinely clinically obtained from patients with AIS, with the exception of those with certain posterior-circulation infarctions that require external ventricular drains [8,9]. Among patients with large hemispheric infarctions, obtaining CSF samples is precluded because of the mass effect and impending risk of herniation. However, the proximity of CSF to the brain parenchyma and other neural products may represent the microenvironment better than blood-based candidates for biomarker development. The use of CSF biomarkers may be particularly beneficial for patients with smaller infarctions, for whom the risk associated with obtaining CSF via lumbar puncture is low. In these scenarios, CSF may be particularly valuable because protein products released as a result of cerebral ischemia may not be otherwise detectable in the peripheral blood because of the low burden of disease. Standard analysis of CSF samples obtained through lumbar punctures for patients with AIS is controversial [10]. Historically, a lack of standardization to minimize the risk of complications has prevented the routine use of lumbar punctures as a diagnostic tool. However, recent advances in proteomics and genomics, as well as updated guidelines and recommendations, have expanded the diagnostic capacity of CSF [11,12]. Another barrier to the clinical use of CSF biomarkers in AIS is that they ideally need to outperform (or demonstrate added value to) computed tomography for diagnosis, which is now obtainable acutely even in relatively resource-poor settings.

In addition to their diagnostic utility, CSF biomarkers for AIS may be beneficial for a variety of other critical clinical needs: determination of stroke etiology (e.g., malignancy), risk of secondary injury (e.g., edema, hemorrhagic conversion, and seizures), risk of recurrence, rehabilitation potential, and outcome. Depending on the specific biomarker, it may also have the potential to inform the response to therapy. Thus, CSF biomarkers may provide both prognostic and predictive enrichment of AIS characterization and facilitate a precision medicine approach to therapy. Importantly, given the proximity of CSF to the AIS microenvironment, CSF biomarkers may be more sensitive than other diagnostic tools and, therefore, easier to identify initially; once these candidates are validated as biomarkers of AIS, targeted assays from the peripheral blood (with high sensitivity for low-abundance proteins) can be subsequently developed and optimized to facilitate detection even in the absence of CSF.

A previous comprehensive systematic review of CSF-based biomarkers in patients with acute brain injury (traumatic brain injury, subarachnoid hemorrhage, AIS, status epilepticus, and postcardiac arrest) included publications between the inception of MEDLINE and June 2021 [13]. Another systematic review and meta-analysis focused on CSF enhancement on imaging [14]. An updated review is therefore needed to explore the relationship between CSF protein biomarkers and patients with AIS.

## 2. Results

### 2.1. Search Results

**Figure 1:**
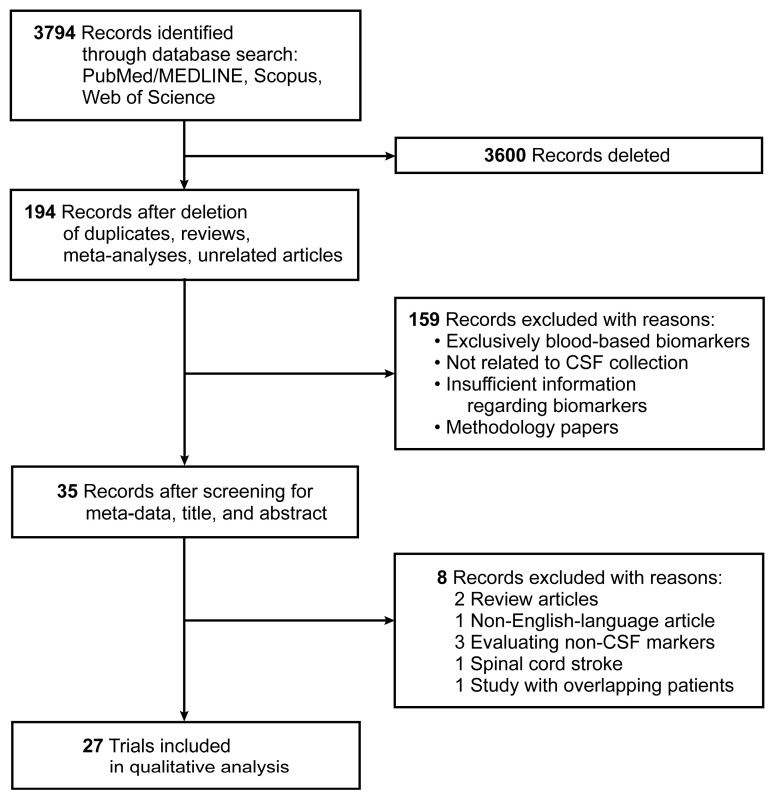
PRISMA diagram showing the flow of evidence of included trials. Used with permission from Barrow Neurological Institute, Phoenix, Arizona.

A total of 3794 articles were identified through the search of three publication databases. After deletions of duplicates, systematic reviews, meta-analyses, and unrelated articles in a preliminary screen using article meta-data, 194 unique articles were identified. A secondary screen of abstracts and titles yielded 35 full-length articles for review, of which 27 met the selection criteria for the systematic review; 22 human studies and five animal studies were analyzed. Figure 1 presents the flow of evidence as a PRISMA diagram.

### 2.2. Characteristics of Included Studies

Of the 22 articles investigating biomarkers in human patients included for analysis (Table 1), 15 trials [15,16,17,18,19,20,21,22,23,24,25,26,27,28,29] compared stroke and non-stroke subjects, and seven studies [30,31,32,33,34,35,36] assessed stroke patients only. Other than one retrospective study investigating markers such as tau-proteins and amyloid-beta [31], the remaining human trials were prospective clinical studies. Further, eight of 22 studies [19,24,25,26,27,28,30,31] evaluated the diagnosis of stroke with CSF biomarkers, whereas 12 studies [15,17,19,21,23,24,25,26,29,33,35,36] used biomarkers to determine stroke progression. The severity of ischemic stroke was studied in 10 articles [15,16,17,18,21,22,23,30,32,33]. Five animal studies were also included in our systematic review (Table 2). One study [37] evaluated a macaque model of CSF. The remaining four studies [38,39,40,41] evaluated a rat model of ischemic stroke. All rat model studies evaluated the progression of ischemic stroke, which was defined as worsening of neurological symptoms in the early course of stroke presentation [42]. In contrast, the macaque model was used to determine the diagnosis and severity of stroke.

### 2.3. Timing and Methodology of CSF Collection

The timing of CSF collection varied within both human and animal studies. A majority of the human studies in this analysis collected CSF in the early hospitalization period. Four studies reported CSF collection immediately at patient admission [19,20,24,36]. Some studies evaluated CSF biomarkers after the first day of routine management, measuring longer-term elevations of biomarkers. A subset of studies also measured CSF biomarkers at various time points in patients to document longitudinal associations of how certain markers changed throughout the natural course of stroke [15,17,24]. All samples were collected by lumbar puncture following standard clinical protocols. In animal studies, CSF was collected immediately after the induction of stroke in the four included studies. In one study [39], CSF was collected three days after the induction of stroke. In all studies using rats, stroke was modeled using middle cerebral artery occlusion (MCAO). After meticulous MCAO, CSF was collected by puncturing the cisterna magna in rats, requiring sacrifice of the animal. This methodology was selected due to ease of accessibility and the provision of adequate CSF volume for various assays necessary. In the macaque model, CSF was obtained seven days after MCAO, immediately prior to animal necropsy.

### 2.4. Biomarkers Identified

Table 3 summarizes unique biomarkers identified in our evaluation of the literature. The three most common CSF markers observed in the literature included S100B, an astrocyte-specific marker expressed in cells that envelope blood vessels in the brain, which was evaluated in four studies, and the two inflammatory markers tumor necrosis factor-alpha (TNF-α) and interleukin (IL) 6, which were each evaluated in four studies. Another commonly reported candidate was free fatty acids, a measure of lipolysis and cell destruction. S100B was found to be correlated with the severity of stroke, defined by neurological symptoms and infarct volume, and patient functional outcome at discharge in multiple human studies as measured by the modified Rankin Scale. Free fatty acids also correlated with NIHSS at discharge and observed final stroke volume as measured by magnetic resonance imaging [34]. In one study of 119 patients, free fatty acids measurements were able to differentiate cardioembolic versus non-cardioembolic ischemic strokes with an area under the curve of 0.801 (95% confidence interval 0.747–0.864) [24]. The strength of the correlation in inflammatory markers was weaker compared to other markers, such as S100B and fatty acids. Although several studies reported the elevation of TNF-α and nitric oxide in stroke, these factors were not independent predictors of outcome or severity [18]. IL-6 was found to be elevated in severe strokes (defined as NIHSS scores 21–42) and a residual of chronic (>1 week) changes after strokes [18,23].

**Figure 2:**
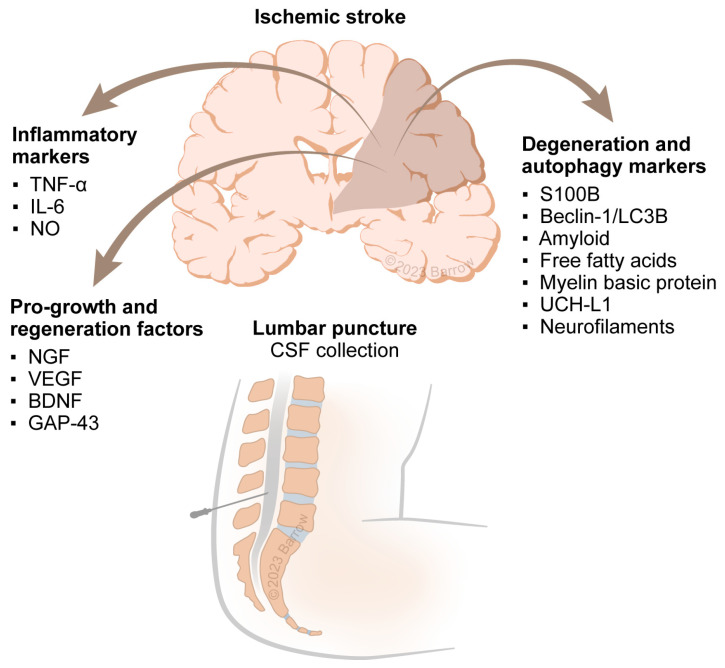
Classes of markers observed in acute ischemic stroke. Abbreviations: BDNF, brain-derived neurotrophic factor; CSF, cerebrospinal fluid; IL-6, interleukin 6; GAP-43, growth-associated protein 43; NGF, nerve growth factor; NO, nitric oxide; TNF-α, tumor necrosis factor-alpha; UCH-L1, Ubiquitin carboxy-terminal hydrolase L1; VEGF, vascular endothelial growth factor. Used with permission from Barrow Neurological Institute, Phoenix, Arizona.

Figure 2 shows the three broad classes of markers that were identified. Pro-growth and regenerative markers, including vascular endothelial growth factor, nerve growth factor, and brain-derived neurotrophic factor [28], were found to be substantially decreased in AIS. Growth-associated protein 43, a cytoplasmic protein responsible for axonal regeneration, was found to be increased two weeks after ischemic insult in one study of 28 patients [17]. A second class of biomarkers included degeneration and autophagy markers. In one study of 37 stroke patients and 21 controls [29], the autophagy markers BECLIN-1 and LC3B were found to be correlated with initial NIHSS scores and functional outcomes defined by modified Rankin Scale scores and improvement in NIHSS scores at three months. A third class of biomarkers indicative of cell-related damage was identified. In two studies [22,32], myelin basic protein, a protein that extravasates out of myelinated sheaths after damage, correlated with stroke location, specifically infarctions localized near white-matter tracts, and clinical stroke severity at admission.

Four human studies investigated a large volume of markers in multiplexed assays [19,20,26,37]. Two of these evaluated circulating microRNA signatures, including Let-7e and miR-338, which influence cell death and apoptosis through the MAPK pathways [43]. The macaque model study by Stevens et al. investigated more than 4000 proteomic markers associated with the clinical severity of stroke and offered a diagnostic value for acute stroke [37]. A rat study investigating multiplexed metabolomic signatures (methylamine, xanthine, pyridoxamine, L-M-monomethyl arginine, glutamine, and histidine) demonstrated the ability to differentiate between infarcted animal models compared to controls [38]. Notably, advanced techniques using metabolomics and proteomics were studied using only animal models. MicroRNA signatures were evaluated exclusively in human trials.

## 3. Methods

### 3.1. Search Process and Article Selection Criteria

A systematic review was performed adhering to the Preferred Reporting Items for Systematic Reviews and Meta-Analysis (PRISMA) guidelines [44] in March 2022. This review was not registered; however, a complete a priori search protocol is available in the Supplementary Materials [15,16,17,18,19,20,21,22,23,24,25,26,27,28,29,30,31,32,33,34,35,36,45]. Articles from PubMed, Web of Science, and Scopus were searched using predefined MeSH terms: (CSF OR (“cerebrospinal fluid”)) AND (stroke OR ischemia OR hemorrhage) AND (biomarker OR marker). These selection criteria included all animal and human studies evaluating CSF biomarkers to diagnose or prognosticate outcomes in patients with AIS. If studies examined both serum and CSF biomarkers, only markers exclusive to the CSF were considered. If these data were not present, articles were excluded. Only full-length articles available in English were considered. For human trials, the acquisition of CSF was constrained to lumbar punctures. Meta-analyses, systematic, and nonsystematic reviews were excluded from the analysis, though they were used for reference matching.

### 3.2. Outcomes Assessed

Various metrics evaluating biomarker utility were considered. For studies testing the diagnostic value of specific CSF markers, metrics including the specificity, sensitivity, negative and positive predictive value, area under the curve, and diagnostic accuracy were considered. Additionally, effect sizes and correlations were evaluated. For studies evaluating the value of biomarkers to prognosticate outcomes, odds ratios and risk ratios were considered and planned to be tabulated. Prognostication was considered for several outcomes, including hospitalization length of stay, delay in functional recovery, need for extended rehabilitation, and cognitive impairment after ischemic stroke. Severity of stroke was assessed via the National Institutes of Health Stroke Scale (NIHSS). Functional recovery was assessed using the modified Rankin Scale. Because of the limited sample size and substantial study heterogeneity present in trials, a meta-analysis was not attempted.

### 3.3. Quality Appraisal of Studies

The quality of studies was assessed for bias using well-established bias assessment tools. For nonrandomized trials, the Newcastle Ottawa Scale was used to validate the study design and account for bias measures [45]. For randomized trials, the Cochrane risk-of-bias tool was used [46]. Bias assessment was performed after study inclusion was determined by authors A.N. and O.A.

## 4. Discussion

### 4.1. Importance of AIS Biomarkers

Time is of the essence when evaluating patients with AIS. Cerebral ischemia causes dysfunction in autoregulatory mechanisms and upregulation and release of prothrombotic molecules that may further impair the regional microcirculation [47]. This dysregulation creates a cycle of more ischemia in nearby regions, with loss of cell membrane integrity, causing cellular content extravasation into the extracellular space. These cellular products vary in concentration based on infarct size, time from infarct, individual characteristics (e.g., resilience to ischemia and robustness of collateral circulation), and cell type, thus forming the basis of precision-biomarker applications. Currently, eligibility for treatment of AIS with intravenous thrombolysis and endovascular thrombectomy relies on establishing the time of symptom onset, usually through a rapid history and neuroimaging with computed tomography [48]. Occasionally, magnetic resonance imaging is involved in the early assessment of suspected stroke [49]. More accessible, less cost-prohibitive, and potentially complementary biomarkers could facilitate speedier diagnoses, inform stroke etiology, quantify pharmacodynamic response profiles, and identify new druggable targets to guide drug development to improve long-term outcomes, survival, and precision medicine.

### 4.2. Biomarker Types and Effect on AIS

Broadly, seven types of biomarkers have been categorized through the *BEST (Biomarkers, Endpoints, and other Tools)* resource, a joint handbook designed by the National Institutes of Health and the Food and Drug Administration [50]. These biomarkers include susceptibility and risk, diagnostic, monitoring, prognostic, predictive, response, and safety biomarkers. In the context of AIS, susceptibility and risk biomarkers, such as blood pressure measurements or cholesterol levels, help identify patients who may be likely to develop stroke [51,52]. In this literature review, multiple CSF-based diagnostic, monitoring, prognostic, and predictive biomarkers are presented, which aid in the diagnosis of AIS, determination of AIS severity, and possible discharge functionality of AIS patients. Response and safety biomarkers are used to evaluate the efficacy and safety of treatment. Given the invasive nature of CSF acquisition through external ventricular drains or lumbar puncture, susceptibility or risk markers and diagnostic markers are unlikely to be cost effective. However, prognostic and monitoring-based biomarkers may be used as prognosticators for patients with severe symptoms. More research is also needed for response and safety biomarkers, given that no studies in our systematic review of the literature measured response to specific or targeted therapies.

### 4.3. CSF as a Solute for Biomarkers

When choosing a biological fluid for sampling, several variables should be considered. Blood testing is less invasive and more accessible than CSF testing, which requires a lumbar puncture [53]. However, when potential biomarkers cross the blood-brain barrier (BBB), they may no longer be intact or may present at low concentrations in blood, making uninformed quantification challenging [54]. The BBB plays a critical role in mediating the release of neuronal and glial cellular constituents into the systemic circulation through blood [55]. Due to this mediation, interpreting serum biomarkers can be difficult because BBB fragility or tissue damage can present with varying amounts of inflammatory products. The vast volume of blood and extracellular fluid can also effectively dilute biomarkers compared with the relatively smaller volume of CSF, reducing the biomarker concentrations as well as the testing time window [54]. However, given advances in proteomic technologies, specific markers identified and validated as useful from the CSF can subsequently have low-abundance assays optimized for detection. In several malignancies and obstetric conditions, proteins at nanoliter concentrations are now detectable in the peripheral blood using novel assays and developing technology [56].

That being said, although blood is a more readily accessible biological fluid, CSF receives degradation productions directly, mirroring biochemical processes in the brain, making it an ideal fluid to assess neurological diseases at an earlier onset [57]. It is possible that biomarkers from the CSF will not translate to the peripheral blood. Nonetheless, the lack of ease in obtaining CSF samples cannot be ignored and is a limitation, especially in resource-poor environments [54].

### 4.4. Leveraging Pathophysiologic Pathways for Biomarker Quantification

Activation of the inflammatory response is a crucial component in the pathophysiology of stroke progression and recovery. In the hyperacute window after ischemia, resident microglia are recruited via various reactive oxygen species and cytokines, such as TNF-α, IL-6, and IL-1β, from localized cellular damage [58]. This localized inflammation contributes to systemic inflammation, where recruitment of systemic leukocytes, monocytes, and T-cells amplify the inflammatory cascade through enzymes such as nicotinamide adenine dinucleotide phosphate oxidase and myeloperoxidase, resulting in a breakdown of the BBB, cerebral edema, and neuronal death [58]. In our systematic review, several inflammatory markers were identified as diagnostic in AIS, as well as grading the severity of patient outcomes. In the study by Beridze et al., 95 patients with ischemic stroke underwent a lumbar puncture during early hospitalization, with CSF evaluation of TNF-α, IL-6, lipoperoxide radical, and nitric oxide [18]. These markers were all markedly elevated compared to 25 non-stroke control patients (all *p* < 0.05). Certain markers were more prognostic of severe stroke, thresholded at an NIHSS score of 21. For example, TNF-α was elevated two- to three-fold in severe and mild-to-moderate stroke, whereas IL-6 had a nearly 30-fold increase in severe stroke and only a 10-fold increase in mild-to-moderate stroke. Vila et al., in 2000, demonstrated in a study of 83 patients that elevated IL-6 at 48 h after stroke was associated with significantly greater risk of clinical progression and worsening prognosis [36]. Although neuroinflammation notoriously plays a dual role, unchecked or prolonged inflammation driven by cytokines like IL-6 is associated with poor prognosis.

A key element of stroke disability involves neuronal death through multiple pathways, including autophagy, apoptosis, and necrosis. Prolonged ischemia drives these processes of neuronal death and also contributes to activating systemic lymphocytes to clear debris from damaged cells. In our review, we uncovered early and delayed evidence of cell death [59]. Markers of autophagy, including Beclin-1 and LC3B, were shown to be significantly elevated in ischemic stroke. Additionally, free fatty acids, a sign of acute cell rupture and membrane destruction, were also found to be elevated [60].

### 4.5. Animal Models of Ischemic Stroke

In our systematic review, the predominant models of acute ischemic stroke were rat models of MCAO. The MCAO model of ischemic stroke has been shown to precipitate a biochemical cascade in brain parenchyma similar to human ischemic stroke [61,62]. These models closely replicate the cerebral vasculature and physiology of humans, in addition to other logistical advantages, including a moderate body size optimal for physiological monitoring, brain size allowing for postexperimental fixation, and reproducibility [63]. Although less commonly used, other models included mice, rabbits, and macaques [64].

### 4.6. Future Directions

Although individual CSF biomarkers have demonstrated the feasibility of diagnosing stroke, determining clinical progression, grading severity, and prognosticating outcome, the future of biomarker-based advancements include multiplexed assays, the use of machine-learning approaches, and point-of-care diagnostics. Recently, multiplexed assays have allowed for the measurement of multiple biomarkers at once. These assays include the use of multiplexed proteomics, metabolomics, and measurement of microRNA fragments [65]. In general, multiplexed assays may be preferred due to the possible reduction in reagents used, reduced cost to the patient and institution, reduced time, and the ability to endotype several pathways simultaneously (e.g., inflammation and cell death) to facilitate risk-stratification and guide therapy. The data generated from multiplexed assays also allow for the use of machine-learning approaches to better diagnose and determine the desired outcomes for patients. Recent studies have demonstrated the ability to rapidly diagnose various acute conditions with multiple serum markers combined with patient features identified from the electronic medical record [66,67]. This approach was associated with far greater specificity and sensitivity than any one biomarker alone. Another important area of innovation relies on improved point-of-care diagnostic technologies, which allow for assays to be conducted near the bedside. In the context of analyzing CSF, the present standard of care requires samples of fluid to be submitted to a laboratory for analysis. Recent studies have demonstrated how microfluidic chambers have been constructed to analyze proteins in serum [68] and CSF [69] for different pathologies. Importantly, in addition to diagnostic and prognostic utility, CSF biomarkers for AIS can be developed for other currently unmet clinical needs: determination of stroke etiology (e.g., malignancy), risk of secondary injury (e.g., edema, hemorrhagic conversion, and seizures), risk of recurrence, rehabilitation potential, and more nuanced cognitive outcomes. As mentioned earlier, specific or targeted biomarkers may also have the potential to inform response to therapy.

### 4.7. Limitations

This systematic review has some notable limitations. First, the biomarkers identified in this study are members of highly complex and multifaceted pathways involved in several underlying pathobiological networks. This complexity makes it difficult to ascertain the unique function of any specific biomarker. Diagnostic reporting for various markers was limited in terms of model discrimination and calibration, including a lack of information regarding specificity, sensitivity, and area under the curve. This limitation prevented us from comparing the diagnostic efficacy of biomarkers via a pooled meta-analysis of specificity and sensitivity or a meta-analysis of diagnostic odds ratios. CSF collection timing was variable. This timing is an important consideration in terms of the predictive, prognostic, and pharmacodynamic utility of biomarkers as well as the ability to meaningfully compare biomarkers. In some patients, CSF was collected in the immediate admission period, allowing for the assessment of acute elevations in certain biomarkers. In contrast, other biomarkers were evaluated days after the initial vascular insult. Given that lumbar punctures are not currently routinely clinically indicated for patients with ischemic stroke after an acute assessment, such biomarkers, though statistically significant, will need to outperform or complement existing clinical and radiographic parameters to have true clinical significance. One advantage of developing these biomarkers is the granular pathobiological information available to endotype AIS, including active pathways that may not only inform progression, prognosis, and treatment-response but also shed light on underlying stroke etiologies that would change management (e.g., distinguishing cardioembolic stroke from atrial fibrillation vs. an underlying malignancy). Another limitation that should be considered is the etiology of stroke (e.g., lacunar vs. non-lacunar strokes). Given the varying pathology of lacunar strokes and differences in the prognosis and clinical features associated with lacunar versus non-lacunar strokes, biomarkers for different stroke etiologies may be different and have varying efficacy, which should be investigated in future studies [70].

## 5. Conclusions

Given the profound burden of AIS on patients and the medical system at large, tools that aid in its early detection and accurate prognostication are vital to improving the quality of care. Various blood-based markers have been previously studied, yet CSF is a relatively understudied biological target that has the potential to offer a closer snapshot of neurological pathology, given its proximity to the diseased microenvironment. In this study, we performed a systematic review of 22 human and five animal studies that sought to evaluate various biomarkers present in CSF to diagnose, prognosticate, and monitor patients with AIS. Our study demonstrates that the following offer prognostic and diagnostic value: inflammatory markers, such as TNF-α and IL-6; autophagy and cell-death proteins, such as SB100 and BECLIN-1; and free fatty acids. Additionally, novel multiplexed protein and microRNA assays offer a new realm of possibilities, especially when augmented with machine-learning methods to predict patient outcomes. Despite these possibilities, we highlighted the need for more research to identify response and safety biomarkers. Additionally, intrinsic safety considerations and limitations to CSF acquisition make the adoption of CSF-based biomarkers difficult to generalize. Nonetheless, an important potential benefit of CSF biomarker studies is that they may facilitate identification and validation of key targets reflective of disease pathophysiology/microenvironment, response to therapy, stroke etiology, secondary injury risk, and progression. In turn, such studies can inform the optimization of targeted low-abundance assays from peripheral but more practical biofluid samples such as plasma.

## Figures and Tables

**Table 1:** Human studies investigating cerebrospinal fluid biomarkers for ischemic stroke.

Author, Year	Study Location	No. of Subjects	Time CSF Collected for Stroke Patients	Study Design	Biomarker Tested	Associated Characteristic	NOS	Study Outcomes
Vila et al., 2000 [36]	Spain	81 stroke	At admission	Prospective clinical trial	Inflammatory markers (IL-6 and TNF-α)	Progression	6	Elevated IL-6 (>6.3 pg/mL) was an independent risk factor of progression/worsening 48 h after admission. TNF-α was elevated in stroke but not independently significant.
Beridze and Shakarishvili, 2006 [23]	Georgia	58 stroke, 15 control	N/A	Prospective	Proinflammatory cytokines IL-1b, IL-6, TNF-α and anti-inflammatory cytokine IL-10	Severity, progression	8	IL-6 levels were considered a stable prognostic indicator of clinical course of disease; only marker statistically significant after 1 wk.
Brouns et al., 2008 [25]	Belgium	85 stroke, 51 control	Within 24 h of stroke onset	Prospective clinical trial	Lactate	Diagnosis, progression	9	Lactate in CSF correlated with stroke evolution in 72 h and patient outcomes at 3 months (mortality, poor outcome (mRS >3)), validated by multivariate models.
Petzold et al., 2008 [27]	UK	33 stroke, 20 control	Early hospitalization	Prospective clinical trial	S100B, ferritin, and NfH SMI35	Diagnosis	9	Elevated S100B and ferritin in patients with ischemic stroke compared to controls. NfH levels were not elevated.
Brouns et al., 2010 [22]	Belgium	89 stroke, 35 control	Mean of 8.7 h after onset	Prospective study	MBP, GFAP, S100B, and NSE	Severity	7	MBP was a marker for infarct location. GFAP and S100B correlated with stroke severity and outcome.
Beridze et al., 2011 [18]	Israel	95 stroke, 25 age-matched controls	Early hospitalization	Prospective clinical trial	Acute phase reactants (IL-1, IL-6, IL-10, TNF-α, NO_2_, NO, LOO)	Severity of stroke	9	All acute phase reactants were generally elevated. NO_2_ and IL-6 were independently predictive of severe stroke symptoms. LOO and TNF-α were also elevated in univariate analysis but not significant in multivariate analysis.
Kaerst et al., 2013 [31]	Germany	18 stroke	Varied from day of ischemic event to several weeks after	Retrospective study	s t-tau, p-tau and Aβ42	Diagnosis	5	Increase in CSF biomarkers depended on size and duration after event; however, even small infarct area led to increased CSF tau levels.
Ke and Zhang, 2013 [28]	China	50 stroke, 30 control	Prior to medication	Prospective clinical trial	HIF-1α, VEGF, NGF, and BDNF	Diagnosis	7	HIF-1a and NGF levels were significantly reduced in stroke patients compared to controls. VEGF and BDNF were unchanged.
Hjalmarsson et al., 2014 [32]	UK	20 stroke	5–10 days after stroke	Prospective study	NfL, T-tau, MBP, YKL-40, and GFAP	Severity	5	T-tau, MBP, YKL-40, and GFAP increased in stroke, and they correlated to clinical stroke severity. However, only NFL was found to be a marker of degree of white-matter lesion.
Sørensen et al., 2014 [19]	Denmark	10 stroke, 10 control	At admission	Prospective study	miRNAs	Diagnosis	8	Two miRNAs (let-7c and miR-221-3p) were upregulated in relation to stroke. Some miRNAs occurred exclusively in the CSF, including miR-523-3p, which was detected in 50% of stroke patients but was completely absent in controls.
Li et al., 2015 [29]	China	37 stroke, 21 control	Early hospitalization	Prospective clinical trial	Autophagy markers (BECLIN1, LC3B)	Progression	9	Demonstrated that BECLIN1 and LC3B were highly correlated with infarct volume and NIHSS scores and moderately correlated with functional outcome (mRS).
Peng et al., 2015 [26]	China	28 stroke, 12 control	Acute stage (11), subacute stage (9), recovery (8)	Prospective clinical trial	MicroRNA markers via PCR (let-7e and miR-338)	Diagnosis, progression	7	Elevated miR-338 was observed in the subacute phase of AIS patients (vs. control) but returned to normal levels in recovery. Elevated let-7e levels were found in all levels of stroke (acute, subacute, and recovery). Let-7e had an AUC of 0.86 for diagnosis of stroke, whereas miR-338 had an AUC of 0.63.
Sun et al., 2015 [24]	China	41 stroke, 78 control	At admission and 12, 24, 48 h postadmission	Prospective clinical trial	FFA levels	Diagnosis, progression	8	Good diagnostic value for cardioembolic vs. non-cardioembolic stroke, correlated for infarction volume and NIHSS scores. A two-fold increase of FFAs compared with the baseline values began 12 h after admission, reaching peak values at 24 h and returning to admission values by 48 h.
De Vos et al., 2017 [30]	Belgium	50 stroke	Mean of 8.7 h after onset	Prospective study	Neurogranin and tau	Diagnosis, severity	5	Tau was a more promising predictor in CSF. Levels of neurogranin were significantly associated with infarct volume but not stroke severity or long-term outcome.
Duan et al., 2017 [33]	China	252 stroke	Within 24 h	Prospective study	FFA levels	Severity, progression	6	Patients with unfavorable outcomes had significantly elevated FFA levels versus patients with favorable outcomes.
Niu et al., 2017 [34]	China	272 stroke	Within 24 h of stroke onset	Prospective clinical trial	FFA levels	Progression	5	Elevated FFA levels correlated to greater stroke volume and NIHSS score for patients.
Sørensen et al., 2017 [20]	Denmark	21 stroke, 21 control	Upon admission	Prospective study	miRNAs	Progression	9	miR-9-5p and miR-128-3p were significantly higher in CSF of stroke patients compared to controls. miRNAs (miR-9-5p, miR-9-3p, miR-124-3p, and miR-128-3p) were elevated in patients with larger infarcts.
Sandelius et al., 2018 [17]	Sweden	28 stroke, 19 control	Day 0–1, day 2–4, day 7–9, 3 wk, 3–5 mo	Prospective study	GAP-43	Severity, progression	8	In the first 2 weeks, a transient increase was noted.
Pujol-Calderón et al., 2019 [15]	Sweden	30 stroke, 30 control	Day 0–1, day 2–3, day 7–9, 3 wk, 3–5 mo	Prospective study	Serum and CSF NfL and NfH proteins	Severity, progression	9	Both serum and CSF NfL and NfH concentrations reflected neuronal injury after acute stroke. The highest levels were around week 3, and levels decreased after 3–5 months.
Gaber et al., 2020 [21]	Egypt	80 stroke, 28 control	Within 24 h	Prospective trial	FFA levels	Severity, progression	7	Positive correlation with larger infarction volume and significant predictor of all-cause mortality.
Hagberg et al., 2020 [35]	Norway	13 stroke	1 y after stroke	Prospective clinical trial	Amyloid-beta 12	Progression	5	CSF markers 1-year post-AIS were not predictive of neurodegeneration or cognitive decline after 7-year follow-up.
Xiong et al., 2021 [16]	China	105 stroke, 80 control	Day 1 after diagnosis	Prospective study	a2d-1	Severity	8	Level of a2d-1 in large infarct volume was significantly higher than in the medium and small infarct volume groups. Levels were also higher in patients with greater severity.

Abbreviations: AIS, acute ischemic stroke; AUC, area under the curve; BDNF, brain-derived neurotrophic factor; CSF, cerebrospinal fluid; FFA, free fatty acid; GAP-43, growth-associated protein 43; GFAP, glial fibrillary astrocytic protein; HIF, hypoxia-inducible factor; IL, interleukin; LOO, lipoperoxide radical; MBP, myelin basic protein; miRNA, microRNA; mRS, modified Rankin Scale; N/A, not available; NfH, neurofilament heavy chain; NfL, neurofilament light chain; NGF, nerve growth factor; NIHSS, National Institutes of Health Stroke Scale; NO, nitric oxide; NO_2_, nitrogen dioxide; NOS, Newcastle Ottawa Scale; NSE, neuron-specific enolase; TNF, tumor necrosis factor; T-tau, total tau; VEGF, vascular endothelial growth factor.

**Table 2:** Animal studies investigating cerebrospinal fluid biomarkers for ischemic stroke.

Author, Year	Study Location	Animal	No. of Animals	Time CSF Collected for Stroke Group	Study Design	Biomarker Tested	Associated Characteristic	Study Outcomes
Tanaka et al., 2008 [41]	Japan	Rats	13 sham, 10 stroke	Immediately after stroke onset	Controlled animal model	S100B protein	Progression	Strokes induced by photochemical MCA occlusion. Presence of S100B in CSF measured over time for stroke vs. sham animals. SC100B significantly increased in stroke group after occlusion and remained elevated up to 48 h.
Liu et al., 2010 [40]	USA	Rats	9 control, 21 stroke	Sequential collection 6 h after stroke induction	Controlled animal model	UCH-L1 protein	Progression	MCA occlusion performed for 30 min or 2 h. UCH-L1 expression increased for 6 h but returned to sham-comparable levels after 1 d for 30-min occlusion group but remained significantly elevated for 5 d for 2-h occlusion group.
Huan et al., 2016 [38]	China	Rats	10 sham, 10 stroke	3 h after induction of stroke	Controlled animal model	Metabolomics (methylamine, xanthine, pyridoxamine, L-M-monomethyl arginine, glutamine, histidine)	Progression	After MCA occlusion, CSF samples were obtained from the cisterna magna. Compared to sham, methylamine, xanthine, and pyridoxamine levels were significantly greater in MCA-occluded rats. Other markers were significantly less compared to sham.
Brégère et al., 2017 [39]	Switzerland	Rats	63 stroke	3 d after stroke induction	Controlled animal model	Doublecortin	Progression	MCA occlusion performed at different time periods, investigating impact of ischemia on neurocognitive development. Doublecortin levels sharply increased 3 d after injury and sustained elevated levels.
Stevens et al., 2019 [37]	USA	Macaque	15 stroke, 4 control	7 d after stroke onset	Controlled animal model	Proteomic signatures (4000+ unique proteins)	Diagnosis, severity	Identified various neuroprotective proteins and pathological proteins via hierarchical clustering and principal component analysis.

Abbreviation: MCA, middle cerebral artery.

**Table 3:** Unique biomarkers observed in the literature.

Biomarkers	Animal or Human Study	No. of Studies	Function or Physiologic Indication
S100B	Animal and Human	4	Astrocyte-specific marker expressed in cells that envelope blood vessels in the brain
General inflammatory markers (TNF-α, IL-6, NO)	Human	4	Inflammatory
Beclin-1	Human	1	Autophagy and cell destruction
LC3B	Human	1	Autophagy and cell destruction
Amyloid (tau, amyloid-beta 12)	Human	3	Neurodegeneration markers
Neurofilament	Human	1	Intraneural cytoplasmic structural proteins
Neurogranin	Human	1	Calmodulin-binding protein expressed in dendritic spines and participated in the protein kinase C signaling pathway
GAP-43	Human	1	Cytoplasmic protein responsible for axonal regeneration after insult
miRNA	Human	2	Variable
GFAP	Human	2	Glial cell marker
MBP	Human	2	Protein responsible for adhesion of the cytosolic surfaces of multilayered compact myelin
Free fatty acids	Human	4	Markers of lipolysis, indicating cell destruction and damage
Ferritin	Human	1	Intracellular protein that binds and releases iron in a concentration-regulated fashion
HIF-1a	Human	1	DNA-binding complex, transcriptional regulator protein controlling response to tissue hypoxia
VEGF	Human	1	Signaling protein that indicates the need for increased blood vessel growth
NGF	Human	1	Protein that promotes growth of nerves and axons
BDNF	Human	1	Protein expressed that acts on the RAS/ERK pathway to increase synaptic density
Metabolomic markers (methylamine, xanthine, pyridoxamine, L-M-monomethyl arginine, glutamine, histidine)	Animal	1	Variable
Proteomic markers (4000+ unique signatures)	Animal	1	Variable
UCH-L1	Animal	1	Deubiquitinating enzyme protein, present in neurons, indicating neuronal destruction
Doublecortin	Animal	1	Microtubule-associated protein promoting stable neural cytoarchitecture

Abbreviations: BDNF, brain-derived neurotrophic factor; GAP-43, growth-associated protein 43; GFAP, glial fibrillary astrocytic protein; HIF, hypoxia-inducible factor; IL, interleukin; MBP, myelin basic protein; miRNA, microRNA; NGF, nerve growth factor; NO, nitric oxide; TNF, tumor necrosis factor; VEGF, vascular endothelial growth factor.

## Data Availability

All data collected has been included in tables. All other data can be made available upon reasonable request to the corresponding author.

## Supplementary Materials

The following supporting information can be downloaded at: https://www.mdpi.com/article/10.3390/ijms241310902/s1.

## Abbreviations

AIS	acute ischemic stroke
BBB	blood–brain barrier
CSF	cerebrospinal fluid
IL	interleukin
MCAO	middle cerebral artery occlusion
NIHSS	National Institutes of Health Stroke Scale
PRISMA	Preferred Reporting in Systematic Reviews and Meta-Analysis
TNF-α	tumor necrosis factor-alpha
