# Supplementary material for: Cerebrospinal Fluid Biomarkers for Diagnosis and the Prognostication of Acute Ischemic Stroke: A Systematic Review
Source: Int J Mol Sci. 2023 Jun 30;24(13):10902. doi: 10.3390/ijms241310902 (PMC10341720; doi:10.3390/ijms241310902)
Supplement: Supplementary file 1 [file ijms-24-10902-s001.zip › ijms-2244418-supplementary.pdf]

**SUPPLEMENTAL MATERIALS**

**Supplemental A Priori Search Protocol.** Systematic review protocol for cerebrospinal fluid biomarkers in the diagnosis and prognostication of outcomes in acute ischemic stroke

**Objective:**

The objective of this systematic review was to evaluate the diagnostic and prognostic utility of cerebrospinal fluid (CSF) biomarkers in patients with acute ischemic stroke (AIS). The review identifies potential CSF biomarkers that could be used as diagnostic and prognostic tools for AIS.

**Methods:**

Search Process and Article Selection Criteria:

This systematic review follows the Preferred Reporting Items for Systematic Reviews and Meta-Analyses guidelines. The authors searched PubMed/MEDLINE, Web of Science, and Scopus for appropriate articles using predefined MeSH terms: (CSF OR ("cerebrospinal fluid")) AND (stroke OR ischemia OR hemorrhage) AND (biomarker OR marker). The search was conducted in March 2022.

Inclusion Criteria:

- Studies published in English language.
- Full-length original research articles that examine the diagnostic or prognostic value of CSF biomarkers in patients or animal models with AIS.
- Studies that report on CSF biomarkers as the primary exposure or intervention of interest.
- Studies that evaluate biomarkers exclusive to the CSF and not biomarkers that are present in both serum and CSF.
- Studies that report on the following outcomes:
    - Diagnostic value: sensitivity, specificity, positive predictive value, negative predictive value, diagnostic accuracy, area under the curve, effect sizes, and correlations.
    - Prognostic value: odds ratios, risk ratios, effect sizes, and correlations for the following outcomes: hospitalization length of stay, delay in functional recovery, need for extended rehabilitation, and cognitive impairment after ischemic stroke.
    - Studies with patients diagnosed with AIS confirmed by standard clinical and/or imaging criteria.

Exclusion Criteria:

- Studies published in a language other than English.
- Reviews, case reports, commentaries, letters, and editorials.
- Studies that did not evaluate CSF biomarkers or that evaluated biomarkers in both serum and CSF.
- Studies that report on nonspecific or non-CSF biomarkers.
- Studies with overlapping or duplicated data.
- Studies that did not report on any of the outcomes of interest.
- Studies with patients with non-AIS stroke types, such as hemorrhagic stroke or transient ischemic attack.
- Studies with patients with other neurological or systemic diseases that may influence CSF biomarker levels.
- Studies with insufficient information to assess the quality of evidence or risk of bias.

**Outcomes Assessed:**

Various metrics evaluating biomarker utility were considered. For studies testing the diagnostic value of specific CSF markers, metrics including specificity, sensitivity, negative and positive predictive value, area under the curve, and diagnostic accuracy were considered. Additionally, effect sizes and correlations were evaluated. For studies evaluating the value of biomarkers to prognosticate outcomes, odds ratios and risk ratios were considered and tabulated. Prognostication was considered for several outcomes, including hospital length of stay, delay in functional recovery, need for extended rehabilitation, and cognitive impairment after ischemic stroke. Severity of stroke was assessed via the National Institutes of Health Stroke Scale (NIHSS), and functional recovery was assessed using the modified Rankin Scale.

**Quality Appraisal of Studies:**

The quality of studies was assessed using established bias assessment tools. Two authors (A.N. and O.A.) independently assessed the quality of the included studies, and discrepancies were resolved through discussion with a third author (S.R.). The Newcastle-Ottawa Scale was used to assess the risk of bias in nonrandomized studies. This scale assesses the quality of the study based on three criteria: selection, comparability, and outcome. Studies were rated from 0 to 9, with scores of 0 to 3 indicating a high risk of bias, scores of 4 to 6 indicating a moderate risk of bias, and scores of 7 to 9 indicating a low risk of bias. For randomized controlled trials, the Cochrane risk-of-bias tool was used to assess the risk of bias. This tool assesses the risk of bias in the following domains: selection bias, performance bias, detection bias, attrition bias, reporting bias, and other sources of bias. Each domain is rated as having a low, high, or unclear risk of bias. A study is considered to have a low risk of bias if all domains are rated as low risk and a high risk of bias if any domain is rated as high risk.

**Planned Statistical Approaches:**

In the case of a meta-analysis, if data are adequate, analyses are performed using software such as R or Stata. Data are summarized using descriptive statistics, such as mean and standard deviation for continuous variables and frequency and percentages for categorical variables. For studies assessing the diagnostic value of specific CSF markers, receiver operating characteristic curves are generated, and the area under the curve is calculated. The sensitivity, specificity, positive and negative predictive values, and accuracy are calculated. For studies assessing the prognostic value of CSF biomarkers, odds ratios, and risk ratios are calculated where applicable. Effect sizes and correlations are calculated. If there are enough studies with similar methodologies and outcomes, a meta-analysis may be conducted using a random-effects model, and heterogeneity would be assessed using the $I^2$ statistic. Sensitivity analyses and subgroup analyses are also performed as appropriate. Publication bias is assessed using funnel plots and the Egger test. A p-value of $<0.05$ is considered statistically significant.

**Ethics and Dissemination:**

This systematic review used published data, and therefore ethical approval was not required. The findings of this review will be disseminated through publication in a peer-reviewed journal and conference presentations.

**Supplemental Table.** Article quality assessment [1]

| | Selection Criteria (4) | Comparability Criteria (2) | Outcome Criteria (3) | |
|---|---|---|---|---|
| **Author, Year** | **Representativeness, Selection, Ascertainment of Exposure, Outcome of Interest Absent at Presentation** | **Comparability of Cohort (Main, Additional Factor),** | **Assessment of Outcome, Sufficient Follow-Up, Adequacy of Follow-Up** | **NOS Score** |
| Vila et al., 2000 [2] | *** | ** | * | 6 |
| Beridze & Shakarishvili, 2006 [3] | **** | ** | ** | 8 |
| Brouns et al., 2008 [4] | **** | ** | *** | 9 |
| Petzold et al., 2008 [5] | **** | ** | *** | 9 |
| Brouns et al., 2010 [6] | **** | * | ** | 7 |
| Beridze et al., 2011 [7] | **** | ** | *** | 9 |
| Kaerst et al., 2013 [8] | ** | ** | * | 5 |
| Ke & Zhang, 2013 [9] | **** | * | ** | 7 |
| Hjalmarsson et al., 2014 [10] | ** | * | *** | 5 |
| Sørensen et al., 2014 [11] | **** | ** | ** | 8 |
| Li et al., 2015 [12] | **** | ** | *** | 9 |
| Peng et al., 2015 [13] | *** | ** | ** | 7 |
| Sun et al., 2015 [14] | **** | ** | ** | 8 |
| De Vos et al., 2017 [15] | ** | ** | * | 5 |
| Duan et al., 2017 [16] | *** | ** | * | 6 |
| Niu et al., 2017 [17] | ** | * | ** | 5 |
| Sørensen et al., 2017 [18] | **** | ** | *** | 9 |
| Sandelius et al., 2018 [19] | **** | * | *** | 8 |
| Pujol-Calderón et al., 2019 [20] | **** | ** | *** | 9 |
| Gaber et al., 2020 [21] | *** | ** | ** | 7 |
| Hagberg et al., 2020 [22] | ** | * | ** | 5 |
| Xiong et al., 2021 [23] | **** | * | *** | 8 |

Abbreviations: NOS, Newcastle-Ottawa Scale.
One star may be awarded for each NOS criterion: Selection criteria, 4 points possible; Comparability, 2 points possible; Outcome criteria, 3 points possible. The NOS score for each article is the sum of the points.

**Supplemental References**


1. Stang A. Critical evaluation of the Newcastle-Ottawa scale for the assessment of the quality of nonrandomized studies in meta-analyses. *Eur J Epidemiol.* 2010;25(9):603-605.
2. Vila N, Castillo J, Davalos A, et al. Proinflammatory cytokines and early neurological worsening in ischemic stroke. *Stroke.* 2000;31(10):2325-2329.
3. Beridze M, Shakarishvili R. Predicting value of cerebrospinal fluid proinflammatory factors in acute phase of ischemic stroke. *Georgian Med News.* 2006(132):53-57.
4. Brouns R, Sheorajpanday R, Wauters A, et al. Evaluation of lactate as a marker of metabolic stress and cause of secondary damage in acute ischemic stroke or TIA. *Clin Chim Acta.* 2008;397(1-2):27-31.
5. Petzold A, Michel P, Stock M, et al. Glial and axonal body fluid biomarkers are related to infarct volume, severity, and outcome. *J Stroke Cerebrovasc Dis.* 2008;17(4):196-203.
6. Brouns R, De Vil B, Cras P, et al. Neurobiochemical markers of brain damage in cerebrospinal fluid of acute ischemic stroke patients. *Clin Chem.* 2010;56(3):451-458.
7. Beridze M, Sanikidze T, Shakarishvili R, et al. Selected acute phase CSF factors in ischemic stroke: findings and prognostic value. *BMC Neurol.* 2011;11:41.
8. Kaerst L, Kuhlmann A, Wedekind D, et al. Cerebrospinal fluid biomarkers in Alzheimer's disease, vascular dementia and ischemic stroke patients: a critical analysis. *J Neurol.* 2013;260(11):2722-2727.
9. Ke XJ, Zhang JJ. Changes in HIF-1alpha, VEGF, NGF and BDNF levels in cerebrospinal fluid and their relationship with cognitive impairment in patients with cerebral infarction. *J Huazhong Univ Sci Technolog Med Sci.* 2013;33(3):433-437.
10. Hjalmarsson C, Bjerke M, Andersson B, et al. Neuronal and glia-related biomarkers in cerebrospinal fluid of patients with acute ischemic stroke. *J Cent Nerv Syst Dis.* 2014;6:51-58.
11. Sorensen SS, Nygaard AB, Nielsen MY, et al. miRNA expression profiles in cerebrospinal fluid and blood of patients with acute ischemic stroke. *Transl Stroke Res.* 2014;5(6):711-718.
12. Li H, Qiu S, Li X, et al. Autophagy biomarkers in CSF correlates with infarct size, clinical severity and neurological outcome in AIS patients. *J Transl Med.* 2015;13:359.
13. Peng G, Yuan Y, Wu S, et al. MicroRNA let-7e Is a Potential Circulating Biomarker of Acute Stage Ischemic Stroke. *Transl Stroke Res.* 2015;6(6):437-445.
14. Sun GJ, Ding SC, Ling WY, et al. Cerebrospinal Fluid Free Fatty Acid Levels Are Associated with Stroke Subtypes and Severity in Chinese Patients with Acute Ischemic Stroke. *World Neurosurg.* 2015;84(5):1299-1304.
15. De Vos A, Bjerke M, Brouns R, et al. Neurogranin and tau in cerebrospinal fluid and plasma of patients with acute ischemic stroke. *BMC Neurol.* 2017;17(1):170.
16. Duan XX, Zhang GP, Wang XB, et al. Elevated Serum and Cerebrospinal Fluid Free Fatty Acid Levels Are Associated with Unfavorable Functional Outcome in Subjects with Acute Ischemic Stroke. *Mol Neurobiol.* 2017;54(3):1677-1683.
17. Niu Z, Hu H, Tang F. High Free Fatty Acid Levels Are Associated with Stroke Recurrence and Poor Functional Outcome in Chinese Patients with Ischemic Stroke. *J Nutr Health Aging.* 2017;21(10):1102-1106.
18. Sorensen SS, Nygaard AB, Carlsen AL, et al. Elevation of brain-enriched miRNAs in cerebrospinal fluid of patients with acute ischemic stroke. *Biomark Res.* 2017;5:24.
19. Sandelius A, Cullen NC, Kallen A, et al. Transient increase in CSF GAP-43 concentration after ischemic stroke. *BMC Neurol.* 2018;18(1):202.
20. Pujol-Calderon F, Portelius E, Zetterberg H, et al. Neurofilament changes in serum and cerebrospinal fluid after acute ischemic stroke. *Neurosci Lett.* 2019;698:58-63.
21. Gaber S, Ibrahim ElGazzar S, Qenawi M, et al. Free Fatty Acids in CSF and Neurological Clinical Scores: Prognostic Value for Stroke Severity in ICU. *Crit Care Res Pract.* 2020;2020:5808129.
22. Hagberg G, Ihle-Hansen H, Fure B, et al. No evidence for amyloid pathology as a key mediator of neurodegeneration post-stroke - a seven-year follow-up study. *BMC Neurol.* 2020;20(1):174.
23. Xiong X, Zhang L, Li Y, et al. Calcium Channel Subunit alpha2delta-1 as a Potential Biomarker Reflecting Illness Severity and Neuroinflammation in Patients with Acute Ischemic Stroke. *J Stroke Cerebrovasc Dis.* 2021;30(8):105874.
